# Agricultural value of Black Soldier Fly larvae frass as organic fertilizer on ryegrass

**DOI:** 10.1016/j.heliyon.2020.e05855

**Published:** 2021-01-02

**Authors:** Regina Menino, Fernando Felizes, Maria Amélia Castelo-Branco, Paula Fareleira, Olga Moreira, Rui Nunes, Daniel Murta

**Affiliations:** a Unidade Estratégica de Investigação e Serviços de Sistemas Agrários e Florestais e Sanidade Vegetal-Laboratório de Solos, Plantas e Águas, Instituto Nacional de Investigação Agrária e Veterinária, I.P. (INIAV), Av. da República, Quinta do Marquês, 2780-159 Oeiras, Portugal; b Unidade Estratégica de Produção e Saúde Animal, INIAV, I.P., Pólo de Investigação da Fonte Boa, Santarém, Portugal; c EntoGreen - Ingredient Odyssey, Santarém, Portugal; d CIISA, Faculty of Veterinary Medicine, University of Lisbon, Lisbon, Portugal; e CBIOS, Faculty of Veterinary Medicine, Lusófona University of Humanities and Technologies, Campo Grande, Lisboa, Portugal

**Keywords:** Black soldier fly larvae, Organic fertilizer, Pot trial, Perennial ryegrass, Entomocompost

## Abstract

*Hermetia illucens* L., known as Black Soldier Fly (BSF) appear as an opportunity to reuse vegetable by-products, as it is easy to reproduce and can be reared in agricultural side streams, allowing the production of both, animal feed (the larvae, after recycling of the vegetal debris) and soil organic fertilizer (insect frass). Although several organic fertilizers, from long ago, have been used in agriculture, there is yet a paucity of experimental data on the evaluation of the fertilization potential of BSF larvae frass (BSFF). The present study is a contribution to access the agronomic and environmental potential of the BSFF as an organic fertilizer. Within this aim, it was conducted a greenhouse experiment with ryegrass, using seven treatments of BSFF. Under the experimental conditions, the results showed a significant effect of BSFF on the overall ryegrass production, with a steady increase (significant at p ≤ 0.05, as accessed through the Tukey test) up to the treatment with a greater rate of application. In what concerns sustainability of soil productivity, at the end of the experiment, there was also positive indications, namely, a significant increase of OM, P and K, for treatments with higher N endowments, together with a constant increase of dehydrogenase activity, from the control to the higher treatment, which was significant for treatments receiving the higher dose of BSFF.

## 1 Introduction

According to FAO (FAO, 2013) it was estimated that approximately one-third of all food produced for human consumption in the world was lost or wasted each year. This wastage (food scrap) may represent a missed opportunity not only to improve global food security, but also to mitigate environmental impacts and improve resources use from food chains. Thus, the use of vegetable by-products might be a contribution to increase the efficiency of nutrient production.

In their review, Čičková and collaborators (2015) refer to the fact that there are many insects that can be used for processing organic by-products, as they can naturally use it as feed, incorporating the nutrients in their bodies and, at the same time, reduce the amounts of these materials in the environment and turning it into a valuable organic fertilizer (which are likely to replace chemical fertilizers currently used), playing an important role in the recycling of organic matter in nature. Moreover, there is also the advantage of using the insect larvae as animal feed, as a source of protein, or for production of secondary products, such as biodiesel, and biologically active substances (Čičková et al., 2015). The *Hermetia illucens* L., known as Black Soldier Fly (BSF), is one of the best candidates because it can develop well in a large diversity of organic by-products and is also commonly found in rotting fruits and plant residues.

There are few studies with preliminary results from experiments testing the ability of the BSF frass (BSFF) as organic fertilizer. The first experiments were done with basil and sorghum (Newton et al., 2005); the subsequent experiments were with asparagus bean (Anggraeni, 2010), corn (Alattar et al., 2016), onion (Zahn, 2017), lettuce and ryegrass (Kebli and Sinaj, 2017), although inconclusive in terms of consistency as far as concerns the positive effect that is intended with the use of these type of entomocompost as organic fertilizer.

The composition of the BSFF, produced with different substrates, is different and there is a need to investigate how it can affect crops yield (Kagata and Ohgushi, 2012; Salomone et al., 2017; Gold et al., 2018; Nana et al., 2018).

In the present experimental study, we focus on the BSFF obtained from agricultural by-products, to evaluate its agronomic potential as organic fertilizer, both in what concerns crop yield and soil sustainability. To achieve these targets, pot trials were conducted in a greenhouse located in the “National Institute for Agrarian and Veterinarian Research, IP” campus, located in Oeiras-Portugal, between November 2018 and May 2019, using ryegrass as plant test.

## 2 Material and methods

### 2.1 *Black soldier fly (*Hermitia illucens *L.) larvae frass (BSFF)*

The BSF used in this study results from the digestion of vegetable by-products from the agri-food industry (potato – *Solanum tuberosum* – and onion – *Allium cepa*) and it is produced by Entogreen®, in accordance with the Portuguese legislation (Costa et al., 2019). This organic compost was tested for *Escherichia coli*, according to the norm ISO 16649–3:2005 and the result obtained revealed that the counting was less than 0.3 cells.g^−1^ of fresh weight. According to the results obtained for its temperature (28 °C), it was considered mature, and the granulometry was <25mm.

The organic compost was also analysed for selected physic and chemical properties (supplementary materials Table 1). The analyses were carried out according to the methods established by the British Standards Institution (BSI) for the following properties: humidity - EN 13040:2007, and the dry matter (DM) was obtained by difference; pH - EN 13037:2011; electrical conductivity (EC) - EN 13038:2011; organic matter (OM) - EN 13039:2011; total K, Ca, Mg, Na, Cu, Zn, Ni, Cr, and Pb - EN 13650:2001. To determine the N-Kjeldhal (organic N + N–NH_4_^+^), the sample was digested with sulphuric acid, followed by distillation and titration. To determine the inorganic N, it was used potassium chloride extraction, followed by distillation with magnesium oxide, for N–NH_4_^+^, and mustard alloy, for N–NO_3_^-^, and the determination of N–NO_2_ by colorimetry (quantitative determination); organic N was obtained by difference between N-Kjeldhal and N–NH_4_^+^. For the evaluation of total P, Fe, Mn, and B, the material was incinerated at 550 (±25) ºC and submitted to a digestion with a chloridic solution; after this procedure, filtered digested samples were analysed for the mineral content using inductively coupled plasma (ICP). The concentration of Hg was evaluated according to the Portuguese norm PE-005-LQARS/LAP (edition 1 from 2015/01/20). In face of these results, and according to the Portuguese Code of Laws number 103/2015, this compost can be classified as an organic fertilizer.

**Table 1:** Mean values for aerial biomass (g of Fresh and Dry Weight per pot – respectively, FW and DW) of the ryegrass, for each cut, and in the total of the five cuts, in each treatment.

Treatment	Aerial biomass (g per pot)											
	FW	DW	FW	DW	FW	DW	FW	DW	FW	DW	FW	DW
	1^st^ cut		2^nd^ cut		3^rd^ cut		4^th^ cut		5^th^ cut		Total of 5 cuts	
T0	18 c	2,24 ns	6,4 d	1,36 d	7,0 e	1,18 d	6,6 e	1,10 e	4,4 c	0,86 d	**42 d**	**6,7 e**
T25	20 bc	2,38 ns	7,7 c	1,64 c	8,5 d	1,38 cd	8,0 d	1,28 d	5,4 b	1,08 c	**49 c**	**7,8 d**
T50	21 ab	2,48 ns	8,9 b	1,84 bc	9,8 c	1,50 bc	8,7 cd	1,38 cd	6,4 a	1,20 bc	**55 b**	**8,4 c**
T75	20 ab	2,32 ns	9,1 ab	1,88 bc	10,1 bc	1,58 bc	9,4 bc	1,52 bc	6,5 a	1,28 ab	**55 b**	**8,6 c**
T100	21 ab	2,44 ns	9,7 ab	2,14 a	11,1 ab	1,76 ab	10,0 b	1,58 b	6,8 a	1,40 a	**59 ab**	**9,6 ab**
T125	20 ab	2,38 ns	9,9 ab	2,16 a	12,0 a	1,96 a	10,0 b	1,68 ab	6,8 a	1,38 a	**58 ab**	**9,3 b**
T150	22 a	2,46 ns	10,1 a	2,22 a	12,0 a	1,96 a	11,3 a	1,82 a	7,0 a	1,40 a	**62 a**	**9,9 a**
**Mean**	**20 A**		**8,8 C**		**10,1 B**		**9,1 C**		**6,2 C**			
**Mean**		**2,39 A**		**1,89 B**		**1,62 C**		**1,48 D**		**1,23 E**		

FW – fresh weight; DW – dry weight; ns - not significant; means in the same column with the same small letter do not differ significantly (p ≤ 0.05), as judged by the Tukey test; means in the same line with the same capital letter do not differ significantly (p ≤ 0.05), as judged by the Tukey test.

### 2.2 Experimental soil

The soil used in the present experiment was collected in a farm, located in Ribatejo region (Portugal), and was classified as Haplic Fluvisol (ISSS-ISRIC-FAO, 2006). The samples used for the pot trial were air-dried, at room temperature, and sieved to pass a 2mm mesh, and, since the soil was heavy, mixed with sand, in a proportion of 2:1 (soil:sand), using a total weight of 3600 g per pot. A sample of the resulting soil was analysed for selected chemical properties, according to the methods used routinely in the laboratories of the “National Research Institute of Agriculture and Veterinary”, in Oeiras (Portugal).

### 2.3 Greenhouse experiment

A greenhouse pot experiment, using ryegrass (*Lolium multiflorum* Lam.), was set up in order to evaluate the agronomic potential of the BSFF as organic fertilizer, and carried out in cylindrical plastic pots (with 15 cm height, 12.5 cm in diameter at the bottom, and 17 cm in diameter at the top) with a surface area of 226.9 cm^2^, filled with 3600 g “sand:soil” (1:2). The seeds were surface sown in each pot, using a density equivalent to 40 kg.ha^−1^.

The experiment, in a randomized plot design, consisted of seven treatments (six different compost rates plus one control treatment) with five replicates each. The rates of compost were calculated according to the N demand by the crop (assumed as 140 kg N per hectare). Based on this, the treatments were the following: T0, corresponding to the negative control, without compost; and T25, T50, T75, T100, T125, and T150, corresponding, respectively, to 25%, 50%, 75%, 100%, 125%, and 150% of the total demand of N supplied by BSFF. The organic compost was mixed to the soil before sowing.

During the plant growth cycle, the greenhouse temperature was kept between 18 and 25 °C, and the pots were regularly watered, with deionized water, to maintain the soil moisture near to 80% of water holding capacity. The pots were disposed in different places every day, after each watering, to eliminate any influence of the day light, in a randomized way.

The ryegrass was harvested every five week, till inflorescence emergency, in a total of 5 cuts, between November 2018 and May 2019. The cuts were performed on all plants at a distance of about 2 cm from de ground, The biomass and chemical composition were evaluated for each cut.

For plant chemical analyses, after harvesting the plant material, in each cut, the fresh weight (FW) was registered, and then it was washed with deionized water. After this process, it was oven dried at 65 °C for about 48 h, until it reached a constant weight. Finally, dry weighted (DW) was evaluated. The dry tissues were ground to pass 0.5 mm sieve, in a bench mill, for chemical analysis. Plant samples were analysed for N by macro Kjeldahl method. Sub-samples were ashed at 450 °C, digested with HCl and analysed for P, K, Ca, Mg, Na, B, Zn, Cu, Fe, and Mn. Phosphorus was determined by spectrophotometer and the other nutrients by ICP-OES.

At the end of the experiment, soil samples were chemically analysed for those properties, so as for soil dehydrogenase activity (DHA). The analysis was not performed in all the treatments, due to the high number of samples and the limitations of the budget, so it was necessary to choose a reduction in treatments, selecting only the most representative ones for the purpose, that were T0, T50, T100, and T150.

DHA was determined by the reduction of 2,3,5-triphenyltetrazolium chloride (TTC) to thiphenyl formazan (TPF), using a modification of the method described by Casida et al. (1964). Three hundred microgram aliquots of soil were suspended in 200 μl of l M Tris-HCl (pH 7.5). After addition of 100 μl 3% TTC, the samples were vortexed and incubated in the dark at 37 °C for 24 h. Following incubation, the TPF produced by each sample was quantitatively extracted by four washes with 500 μl-increments of methanol, each followed by centrifugation. The centrifugation supernatants were combined (final volume 2.3 ml) and colour intensity was measured spectrophotometrically at 485 nm. The amount of TPF was calculated by reference to a calibration curve prepared from TPF standards. Dehydrogenase activity of each soil (expressed as mg TPF per g of dry soil and hour) corresponds to the average of six replicate samples, previously corrected with the mean value of two blank samples, treated exactly the same way but to which no TTC was added prior to incubation. Since tetrazolium salts are light sensitive (Tabatabai, 1994), all procedures were performed avoiding direct exposure to light.

### 2.4 Data analysis

The experimental data were analysed for variance by the General Linear Model (GLM) and means separation was performed using Tukey's Honestly Significant Difference (HSD) test at p ≤ 0.05.

## 3 Results and discussions

### 3.1 Plants

The aerial biomass of the ryegrass (Table 1) was significantly lower for the T0 treatment, comparing to the treatments T50 or greater, evidencing the fertilizer potential of the BSFF. The highest yield was always produced in the highest rates of compost used, although not always significantly different from the other treatments where the organic compost was used. These results were fairly consistent, either as regarding the fresh weight (FW) or dry weight (DW), except for the dry weight (DW) in the first cut.

**Figure 1:**
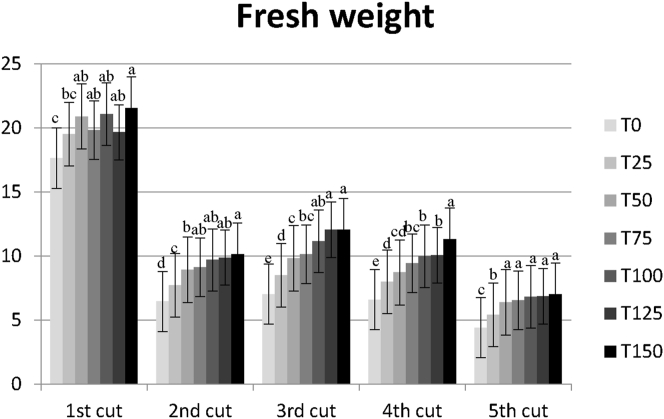
Trend of the aerial biomass obtained in each of the 5 cuts, along the ryegrass cultural cycle, for the fresh (FW). Error bars represent the standard error of the mean (SEM). Columns with different superscript differ significantly: p < 0.05.

**Figure 2:**
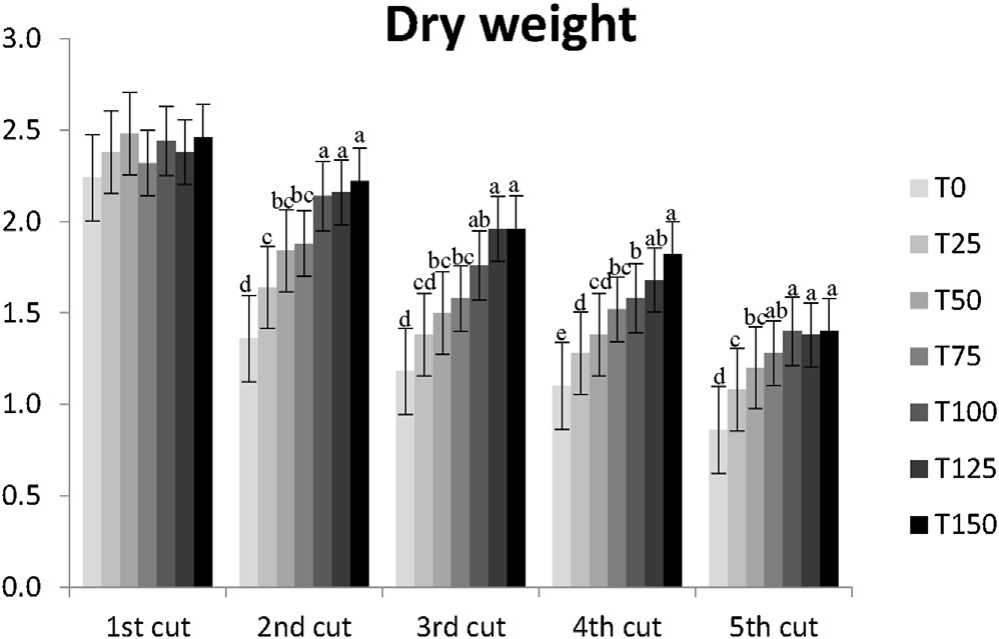
Trend of the aerial biomass obtained in each of the 5 cuts, along the ryegrass cultural cycle, for the dry weight (DW). Error bars represent the standard error of the mean (SEM). Columns with different superscript differ significantly: p < 0.05.

In Figures 1 and 2 it is possible to observe clearly the effect of the increasing BSFF rates on the aerial biomass ryegrass production in the several cuts performed.

For the overall ryegrass production there was a significant effect of BSFF with a steady increase till the treatment T100 (equivalent to 140 kg N ha^−1^). These findings suggest that a higher rate of BSFF could have been tested.

Regarding the mean values for the overall treatments, there was a significant decrease of shoot biomass along the plant cycle. This might have happen because of the immobilization of nutrients in the soil, due to the stimulation of microbial activity, as suggested by the increase in dehydrogenase soil activity (Table 4).

The results for ryegrass shoot biomass of the present experiment are greater than those obtained by Kebli and Sinaj (2017) that have also worked with ryegrass; however these have been obtained with a different frass composition and, eventually, may be due to the number of cuts made in the present experiment. Nevertheless, the positive effect of BSFF on the ryegrass production, increasingly pronounced, is in accordance with the findings of these referred authors, who concluded that it “would be a promising strategy in conventional and/or organic farming”.

For the mean value of biomass production per cut, in the overall treatments, it was observed that the highest yield was obtained in the first cut, and the lowest yield in the last cut, both for FW and DW. This was to be expected, since the plants could use the mineral nutrients available in the beginning, and the BSFF mineralization rate did not match the need of the ryegrass.

The significantly different results, obtained for the chemical analysis of plant nutrients (Table 2) in the different cuts, are caused by the stage of the plant cycle, varying along the time. The results obtained in the present experiment are in accordance with the results reported by Edmeades et al. (1983) and, more recently, by Aganga et al. (2004), who also found different concentrations of the elements, when determined at different stages of growth.

**Table 2:** Nutrients content in the ryegrass dry tissues for each treatment and for each of the five cuts.

Treatment	N	P	K	Ca	Mg	Na	Cu	Fe	Zn	Mn
	g kg^−1^						mg kg^−1^			
1^st^ cut	23 a	6.5 ab	55 a	4.5 c	1.9 c	2.1 b	8.9 a	72 a	27 d	34 c
2^nd^ cut	12 b	7.4 a	43 b	6.8 b	3.2 b	1.7 b	6.3 b	41 b	27 d	59 b
3^rd^ cut	11 c	5.6 b	40 b	8.3 a	4.8 a	2.1 b	5.4 bc	38 bc	35 c	78 a
4^th^ cut	11 c	6.1 b	35 c	8.3 a	4.7 a	3.2 a	4.9 c	36 bc	53 b	80 a
5^th^ cut	11 c	5.3 b	19 d	6.6 b	4.6 a	7.5 a	0.5 d	31 c	66 a	27 d

Means in the same column with the same small letter do not differ significantly (p ≤ 0.05), as judged by the Tukey test.

Comparing the values obtained in the present experiment, for nutrients content, with those published by Jones et al. (1991) for *Lolium perenne*, it can be observed that the N concentration was about half and for the remaining elements (P, K, Ca, Mg, Cu, Zn, and Mn) the values, in general, were greater. The significantly lower value of Mn in the last cut, may be due to the fact that this micronutrient may have been extracted by the plant till a level that it was deficient in the soil, and the plant could not have enough available. It seems that N might acted as a limiting factor and studies evaluating the combination of BSFF with more ready available N (like mineral N) could be interesting.

### 3.2 Soil

The chemical results of the analysis of the experimental “soil:sand” mixture used (Table 3), revealed a neutral pH soil, with medium (Zn), low (N, K, P, Cu and Fe) and very low (OM and Mn) components’ levels.

**Table 3:** Soil chemical analyses.

Treatment	pH H_2_O	OM	Total N	K	P	Cu	Zn	Fe	Mn
		g kg^−1^		mg kg^−1^					
At the beginning of the experiment									
Soil:sand	7.41	4.10	0.01	21	10	0.77	3.02	6.59	3.73
At the end of the experiment									
T0	8.3	7.5 b	0.34	79 b	11 d	0.66	0.47 bc	3.30 a	0.91 bc
T25	8.3	7.4 b	0.44	97 ab	11 d	0.72	0.53 abc	3.29 a	0.98 abc
T50	8.3	8.2 ab	0.31	102 ab	12 cd	0.67	0.46 bc	3.12 bc	0.83 c
T75	8.3	9.0 ab	0.40	97 ab	14 bcd	0.67	0.43 c	3.08 c	0.80 c
T100	8.3	10.0 a	0.53	112 a	15 bc	0.66	0.63 a	3.23 ab	1.05 ab
T125	8.3	9.8 a	0.31	104 ab	16 a	0.71	0.57 ab	3.20 abc	1.05 ab
T150	8.2	10.1 a	0.62	118 a	21 a	0.63	0.46 c	3.31 a	1.11 a

Means in the same column with the same small letter do not differ significantly (p ≤ 0.05, as judged by the Tukey test).

Comparing the initial results for chemical properties, obtained for the initial soil mixture with those obtained after the experiment (Table 3), it is evident an increase on the values obtained for the properties analysed, except for Fe and Mn. This is due to the presence of the organic compost as well as to the plants, because although it was not used organic compost in T0, the presence of the plant roots produced a positive effect on its composition.

The significant increase in OM, K and P, in the present case, was expected, since the frass that was used is very rich in those elements. Zahn (2017), working with onion, to assess the effect of BSFF (from larvae fed with avocado, banana and avocado wastes) predicated that the frass may positively affect plant growth, through the increase of OM, N and P in the soil, although he failed in finding significant effect of BSFF on yield.

In concomitance with plant production, dehydrogenase activity (DHA) increased significantly (Table 4) with increasing amounts of compost used, indicating that the overall soil microbial activity increased. Dehydrogenases are intracellular enzymes that are involved in the redox processes of active microbial cells and have been considered a good measure of the oxidative microbial metabolism in soils (Herman and Maier, 2000).

**Table 4:** Dehydrogenase activity in the different treatments.

Dehydrogenase activity	Treatment			
	T0	T50	T100	T150
μg TPF g^−1^ dry soil	1.257 b	1.800 b	2.380 a	2.598 a

Means with the same small letter do not differ significantly (p ≤ 0.05), as judged by the Tukey test.

**Figure 3:**
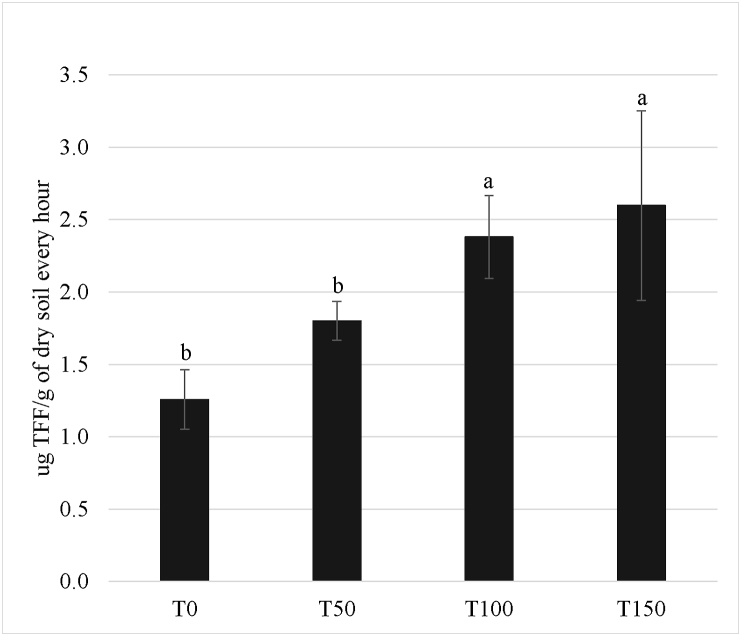
Trend of the dehydrogenase activity in the BSFF rates tested. Error bars represent the standard error of the mean (SEM). columns with different superscript differ significantly: p < 0.05.

In Figure 3 it is possible to observe clearly the trend in the dehydrogenase activity as affected by the BSFF treatments tested for this enzyme.

In terms of sustainability of soil productivity, the organic compost had a positive effect above treatment T100 (equivalent to 140 kg ha^−1^), together with a constant increase of dehydrogenase activity, from the control (T0) to the higher treatment (T150), which was not significant above treatment T100.

## 4 Conclusions

Under the experimental conditions, the results showed a significant effect of BSFF on the overall ryegrass production, with a steady increase until the treatment T100. In what concerns sustainability of soil productivity, at the end of the experiment, there was also positive indications, namely, a significant increase of OM, P_2_O_5_ and K_2_O, above treatment T100, together with a constant increase of dehydrogenase activity, from the control to the higher treatment, which was significant above treatment T100.

The importance of these results justify the need for more experiments, using frass from different origins (different composition), in different edafoclimatic conditions, with different crops, and testing several rates of BSFF, in order to advise its use as organic fertilizer with accuracy in each case. The combination of BSFF with N fertilizers seems also promising.

In the context of a circular economy, its paramount to study the residual effect of BSFF, particularly in soils with low mineral and water retention, so as the most responsive cultures to be considered in crop rotations, so as in monoculture.

## Declarations

### Author contribution statement

Regina Menino, Daniel Murta: Conceived and designed the experiments; Analyzed and interpreted the data; Wrote the paper.

Fernando Felizes, Maria Amélia Castelo-Branco: Performed the experiments.

Paula Fareleira: Performed the experiments; Analyzed and interpreted the data.

Olga Moreira: Conceived and designed the experiments; Contributed reagents, materials, analysis tools or data.

Rui Nunes: Performed the experiments; Analyzed and interpreted the data; Contributed reagents, materials, analysis tools or data.

### Funding statement

This work was supported by PT2020 through the project (POCI-01-0247-FEDER-017675: ENTOVALOR – Insects as an opportunity in residues valorization (2016–2019)).

### Data availability statement

Data included in article.

### Declaration of interests statement

The authors declare no conflict of interest.

### Additional information

No additional information is available for this paper.
