# Supplementary material for: Agricultural value of Black Soldier Fly larvae frass as organic fertilizer on ryegrass
Source: Heliyon. 2021 Jan 2;7(1):e05855. doi: 10.1016/j.heliyon.2020.e05855 (PMC7785954; doi:10.1016/j.heliyon.2020.e05855)
Supplement: Supplementary material - Table 1 [file mmc1.docx]

**Agricultural value of Black Soldier Fly larvae frass as organic fertilizer on ryegrass**

Regina Menino^1^, Fernando Felizes^1^, Maria Amélia Castelo-Branco^1^, Paula Fareleira^1^, Olga Moreira^2^, Rui Nunes^3^, Daniel Murta^3,4,5^

*^1^Unidade Estratégica de Investigação e Serviços de Sistemas Agrários e Florestais e Sanidade Vegetal-Laboratório de Solos, Plantas e Águas, Instituto Nacional de Investigação Agrária e Veterinária, I.P. (INIAV), Av. da República, Quinta do Marquês, 2780-159 Oeiras, Portugal; ^2^Unidade Estratégica de Produção e Saúde Animal, INIAV, I.P., Pólo de Investigação da Fonte Boa, Santarém, Portugal; ^3^EntoGreen - Ingredient Odyssey, Santarém, PORTUGAL;* *^4^CIISA, Faculty of Veterinary Medicine, University of Lisbon, Lisbon, PORTUGAL; ^5^CBIOS, Faculty of Veterinary Medicine, Lusófona University of Humanities and Technologies, Campo Grande, Lisboa, PORTUGAL*

Corresponding author: Daniel Murta (E-mail: daniel.murta@entogreen.com)

Table 1. BSFF characteristics evaluation

| **Properties** | **Value obtained** | **Quality class** |
| --- | --- | --- |
| Dry matter | 85% |  |
| pH (H_2_O) (25 ºC) | 8.61 |  |
| Electrical conductivity | 2.77 mS/cm |  |
| Organic matter | 83.7% |  |
| Total N (Kjeldhal) | 28.1 g kg^-1^ |  |
| Organic N | 25.71 g kg^-1^ |  |
| N-NH_4_^+^ | 2.39 g kg^-1^ |  |
| N-NO_3_^-^ | 86 mg kg^-1^ |  |
| N-NO_2_ | 6.11 mg kg^-1^ |  |
| Total K | 33 g kg^-1^ |  |
| Total P | 15 g kg^-1^ |  |
| Total Ca | 15 g kg^-1^ |  |
| Total Mg | 7 g kg^-1^ |  |
| Total Na | 0.3 g kg^-1^ |  |
| Total Cu | 19 mg kg^-1^ | Class I |
| Total Zn | 137 mg kg^-1^ | Class I |
| Total Ni | <33.3 mg kg^-1^ | Class I |
| Total Cr | <16.7 mg kg^-1^ | Class I |
| Total Pb | <33.3 mg kg^-1^ | Class I |
| Total Hg | <0.006 mg kg^-1^ | Class I |
| Total Fe | 896 mg kg^-1^ |  |
| Total Mn | 149 mg kg^-1^ |  |
| Total B | 25 mg kg^-1^ |  |
| Maturation grade | 28 ºC | IV and V (mature) |
| Granulomety | < 25mm |  |
